# The Development of an Innovative Embedded Sensor for the Optical Measurement of *Ex-Vivo* Engineered Muscle Tissue Contractility

**DOI:** 10.3390/s22186878

**Published:** 2022-09-12

**Authors:** Ludovica Apa, Marianna Cosentino, Flavia Forconi, Antonio Musarò, Emanuele Rizzuto, Zaccaria Del Prete

**Affiliations:** 1 Department of Mechanical and Aerospace Engineering, Sapienza University of Rome, 00184 Rome, Italy; 2 DAHFMO-Unit of Histology and Medical Embryology, Sapienza University of Rome, 00161 Rome, Italy

**Keywords:** contractile force measurements, tissue engineering, optical tracking algorithm, noninvasive measurements, electrical stimulation, 3D *in-vitro* system, skeletal muscle, tissue biomechanics, sensor development

## Abstract

Tissue engineering is a multidisciplinary approach focused on the development of innovative bioartificial substitutes for damaged organs and tissues. For skeletal muscle, the measurement of contractile capability represents a crucial aspect for tissue replacement, drug screening and personalized medicine. To date, the measurement of engineered muscle tissues is rather invasive and not continuous. In this context, we proposed an innovative sensor for the continuous monitoring of engineered-muscle-tissue contractility through an embedded technique. The sensor is based on the calibrated deflection of one of the engineered tissue’s supporting pins, whose movements are measured using a noninvasive optical method. The sensor was calibrated to return force values through the use of a step linear motor and a micro-force transducer. Experimental results showed that the embedded sensor did not alter the correct maturation of the engineered muscle tissue. Finally, as proof of concept, we demonstrated the ability of the sensor to capture alterations in the force contractility of the engineered muscle tissues subjected to serum deprivation.

## 1. Introduction

The development of three-dimensional (3D) engineered tissues represents a valid approach for regenerative medicine, whose aim is to restore the functionality of tissues and organs damaged by age, disease, injuries or congenital defects [1,2]. Tissue engineering is a multidisciplinary approach that involves the combination of living cells with a synthetic bioartificial or natural support to develop substitutes characterized by structural, mechanical and functional properties as close as possible to the native tissue [3,4]. Among all tissues, skeletal muscle represents the most abundant in the human body, amounting to 40–50% of the average weight [5]. It is a metabolically active tissue requiring a constant flow of nutrients and metabolites, provided by an extensive capillary network forming an organized pattern throughout the fibers [6]. The engineering of skeletal muscle *in-vitro* holds promise for the treatment of a variety of muscle diseases [7], including skeletal myopathies such as muscular dystrophy or spinal muscular atrophy [8,9], as well as for the design of *in-vitro* models employed for drug screening or the investigation of phenomena regulating disease onset and progression [10,11,12]. In this context, we developed a 3D engineered skeletal muscle tissue obtained from murine primary cultures without using any scaffold, named *ex-vivo* muscle engineered tissue X-MET [13]. The X-MET shows both morphological and functional characteristics similar to *in-vivo* skeletal muscle. In detail, in our previous work [13], we found that the structure of engineered tissue was constituted by a longitudinal distribution of parallel myotubes surrounded by layers of connective tissue, similar to those observed in a muscle section for a skeletal muscle. In Carosio et al. [13], we also demonstrated the presence of a vessel-like structure that allows for nutrient diffusion and guarantees the culture survival of X-MET. Furthermore, we measured several mechanical parameters, starting from the measurements of the spontaneous contraction [14] to that induce by electric stimulation [13], as well as the mechanical power [15,16]. In short, the possibility of accurately controlling and measuring the functional properties of engineered muscle tissues, such as the contractile force, is a fundamental aspect of tissue engineering allowing for a valid characterization of the tissues before their transplant or modeling for the study of disease alterations. To this aim, the development of new platforms and technologies for assessing the contractile functions of *in-vitro* engineered tissues, also allowing for embedding a series of different measurement sensors, is essential for improving tissue development. Most of the existing methods for the measurement of contractile force are direct. They are based on the use of a high-resolution force transducer coupled to one end of the engineered specimen, while the other end is fixed to the substrate [17,18,19]. These platforms were used to analyze different conditions of the 3D engineered tissues, such as co-cultures [20], cell sources [21,22] and media supplements [23], as well as to perform drug screening [9]. Despite the high accuracy of force measurements, the use of a force transducer only allows for performing endpoint measurements and requires direct contact with the specimen. In fact, usually, the *in-vitro* 3D tissues grow between two anchors, and during the contractility measurement, one end of the construct has to be detached by its anchor and connected to the force transducer, thus inducing possible tissue structure damage and consequent changes in contractility.

To overcome these limitations, noninvasive devices and techniques such as optical methods have been recently proposed for the measurement of the contractile force. Their working concept is mainly based on the measurement of a cantilever or post deflection [24]. Santoso et al. [25] used the muscular thin film (MTF) assay to quantify differences in contractile force between the muscle tissue engineered from the C_2_C_12_ cell line and human muscle tissue. Specific optical programs were used to determine the radius of curvature of each MTF cantilever and calculate tissue stress using a modified Stoney’s equation. Guo et al. [26] fabricated a microelectromechanical system (MEMS) based on a silicone cantilever to test the contraction force of the myofibers grown on them. A detection system was designed to measure the deflection of the cantilever during the myotubes’ contraction. Finally, Nagashima et al. [27] developed a microdevice constituted by micro-posts where the tested tissue was clamped for measuring the contractile force of human skeletal muscle tissue. The contractile force was obtained by the measurement of the tip displacement of the micro-posts through an upright microscope, when the tissue was electrically stimulated. All these methodologies are the most used for the 3D *in-vitro* measurement of muscle tissues’ contractility because of their main advantages, such as the noninvasiveness for the tissue and the possibility of the continuous monitoring of the contractility. On the other hand, they are highly complex to be realized since they require microfabrication facilities or specific fabrication devices. Furthermore, they are not easy to integrate with other measurement systems, for example high-resolution microscopy.

Recently, other emerging technologies have been proposed as alternative ways to measure contractility, such as traction force microscopy, traditionally used for the measurement of cell contractility [28] and now extended to the tissue level [29] and to microelectrode arrays (MEAs) [30], whose working concept is based on the recording of the bioelectrical signal generated by a culture. These methodologies are noninvasive and compatible with high-resolution microscopy, but they present some limitations. First, they do not provide the direct measurement of the contractile force but measure the contraction and relaxation velocities curves, where contractility is usually presented as percentage of the movement [31]. Other limitations are inherent to the setup, being compatible only with specific types of culture (such as those characterized by a low cellular density [32]) and requiring the entire tissue to be placed in direct contact with the device [30].

Within this context, here, we propose the development of an embedded sensor for the continuous monitoring of engineered muscle tissue’s contractility though the use of an optical tracking technique. The sensor core is based on a calibrated pin employed as a standard tissue’s supporting pin whose movements are measured through a noninvasive optical method. The proposed sensor returns the force developed by the 3D *in-vitro* engineered tissue following calibration with a linear actuator and a micro-force transducer. Although the working concept of our sensor, based on the deflection of an elastic pin following the tissue contraction, is similar to those mentioned above [26,27], the proposed sensor provides several advantages in terms of fabrication, applications and integration with the samples. First, our sensor was designed to not alter the engineered tissue’s growth and development and to avoid multiple changes in the tissue’s connections to the external force transducer, thereby being fully embedded in standard culture dishes. In view of this, the sensor can be easily integrated with an optical setup to measure the tissue contraction in a noninvasive manner and to perform continuous measurement for prolonged periods. Finally, the sensor for monitoring the tissue contractility was realized with low-cost materials, its fabrication does not require specific and complex devices, and the production time is quite short.

This sensor was then used to measure the contractile force of five X-METs for five consecutive days to test its noninvasiveness in terms of correct tissue growth. Since the loss of muscle functionality occurs in different compromised conditions, such as traumatic injury, prolonged denervation, acute trauma and aging, here we proposed as *proof-of-concept* the use of this embedded sensor to capture alterations in contractile force in X-MET specimens subjected to alterations in anabolic factors. This condition was obtained by culturing specimens in a serum-free medium enriched with bovine serum albumin (BSA), known to mimic an aged microenvironment. As the main protein of several sera, such as fetal bovine serum and horse serum, albumin was indicated as the most important factor associated with successful attempts to maintain cells in the absence of serum [33,34]. Epidemiological studies indicated that age-related declines in muscle mass and strength (sarcopenia) are associated with decreased levels of growth factors [35,36]. Based on this evidence, we proposed a sensor for discriminating this specific X-MET culture condition whose morpho-functional properties may be influenced by reduced levels of growth factors through the measurement of contractile force.

## 2. Materials and Methods

### 2.1. Sensor Design

**Figure 1:**
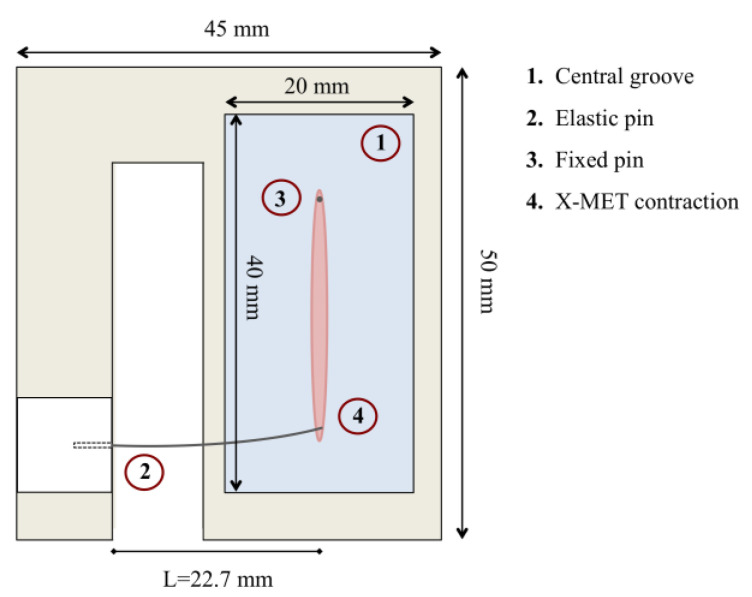
Schematic of the sensor (top view) for the measurement of X-MET contractility.

Figure 1 shows a schematic of the embedded sensor realized for measuring the *ex-vivo* engineered muscle tissue’s contractility during its development. The sensor was designed to guarantee a proper clamping for the growth of the X-MET without altering its correct maturation for at least five days, which represents a crucial aspect for *ex-vivo* engineered tissues, as well as to allow the measurement of tissue contractility based on an optical tracking algorithm. Indeed, the mechanical resistance the two pins exert on the tissue is a crucial stimulus for the growth of muscle-like structures [21,37]. To these aims, the sensor was developed with external dimensions of 50 × 45 × 8 mm to be placed in a 90 mm diameter Petri dish and placed in the incubator during all the growth. The sensor was realized in plexiglass to allow for sterilization by ultraviolet light and comprised a central groove with dimensions of 40 × 20 × 4 mm coated with silicone (Sylgard 184, Dow Corning) to allow for the accommodation of the X-MET. In particular, one end of the specimen was pinned to the silicone substrate, while the other end was linked to a more compliant pin held horizontally with a specific support. Indeed, the base concept of our measurement system is to measure, with an optical tracking algorithm, the deflection of the compliant pin during tissue contraction. The dimensions and the mechanical properties of the elastic pin were chosen according to theoretical considerations [38]. In details, the behavior of the elastic pin was approximated to that of a cantilever subjected to bending. Considering a circular pin with a radius of R = 0.15 mm and a Young’s modulus of *E* = 169 GPa (from the datasheet), we designed the length of the elastic pin *L* to have a deflection of its tip in the range 5–50 μm according to the following equation:

(1) $$k=\frac{3EI}{L^{3}}$$

where *I* is the moment of inertia for a circular pin and *k* is the elastic constant, given from the ratio between the force developed by the tissue and the pin tip displacement. Of note, the range 5–50 μm was chosen taking into consideration the force values usually generated by these tissues during electrical stimulation [15] and those expected to be generated by the treated samples when simulating an aged microenvironment, as suggested in the literature [39,40]. We also considered the needs of the optical tracking method on the basis of different validation tests performed in our previous works [38,41]. The elastic constant result was *k* = 20 N/m for a pin length of *L* = 22.7 mm.

### 2.2. Elastic Pin Calibration

Once the sensor was realized, the elastic pin was calibrated to compute the experimental elastic constant *k*. One end of the elastic pin was fixed to a linear actuator with a resolution of 0.10 μm (NA11B16-T4, Zaber, Vancouver, Canada), and the other end, at the desired length (22.7 mm), was connected to a micro-force transducer with a resolution of 1 μN (AE801, Kronex, Minsk, Belarus). The elastic pin was subjected to 6 different known displacements by the linear actuator, namely, 5 μm, 10 μm, 20 μm, 30 μm, 40 μm and 50 μm. This range of displacements was chosen as that expected from the force range previously reported for a theoretical elastic constant of 20 N/m. The duration of the displacement signals was set at 2 s, and each test was performed 10 times. Custom-made software developed in LabVIEW 2019 allowed for synchronization between the linear actuator displacement and the force signal acquisition.

### 2.3. Experimental System and Stimulation Protocol

All experiments were conducted according to the guidelines of the Declaration of Helsinki and of the Italian National Law about the use of animals for research. The X-MET specimens were obtained according to a protocol similar to that described by Carosio et al. [13]. Briefly, a heterogeneous cell population was obtained by the isolation of skeletal muscle from the hind limbs of a wild-type mouse (WT) using a process of mechanical and enzymatic digestion (gentleMACS Octo Dissociator, MiltenyiBiotec, Bergisch Gladbach, Germany). After 5–6 days in culture, the cells were induced to differentiate using a differentiation medium, and after 2–3 days, the cellular monolayer was delaminated by moving a tip around the peripheral area of the dish. To allow for the complete remodeling of the X-MET in a self-organized cylindrical structure, the delaminated monolayer was clamped in a silicone-coated dish and maintained for three days. After this period, the specimens were clamped in the proposed sensor.

Differentiation medium (DM) that constituted Dulbecco’s Modified Eagle Medium (DMEM), 5% horse serum, 25 mM HEPES, 4 mM L-glutamine, 0.1% gentamicin and penicillin/streptomycin was used for the growth and the maintenance of the X-METs in culture. After demonstrating that the developed embedded sensor did not affect the correct growth of the X-METs, we tested the ability of the sensor to capture alterations in the force contractility of the engineered muscle tissues treated with bovine serum albumin (BSA), known to mimic an aged microenvironment [35,36]. In view of this, four different X-METs were cultured in DMEM enriched with 1% bovine serum albumin (BSA) from the day they were placed in the novel sensor. The contractile properties of the specimens were measured for five consecutive days. This duration was chosen for two main reasons. First of all, the accelerated aging induced with our treatment yielded to unresponsive X-METs after three days in culture. Second, we wanted to demonstrate the capability of our sensor to measure the contractility of the control X-METs and to verify the correct growth and maturation of the tissues over a longer period.

**Figure 2:**
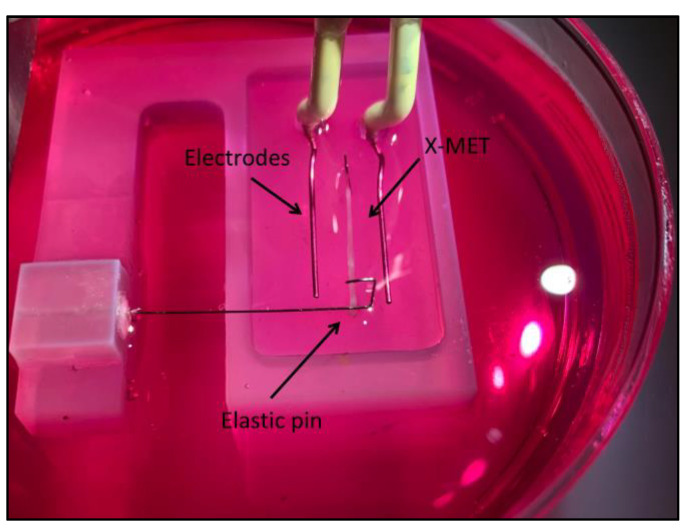
Picture of the sensor integrated with the platinum electrodes for the electrical stimulation of the tissue. One end of the specimen is pinned in the silicone substrate, and the other end is linked to the elastic pin. The entire sensor is placed in a 90 mm diameter Petri dish.

**Figure 3:**
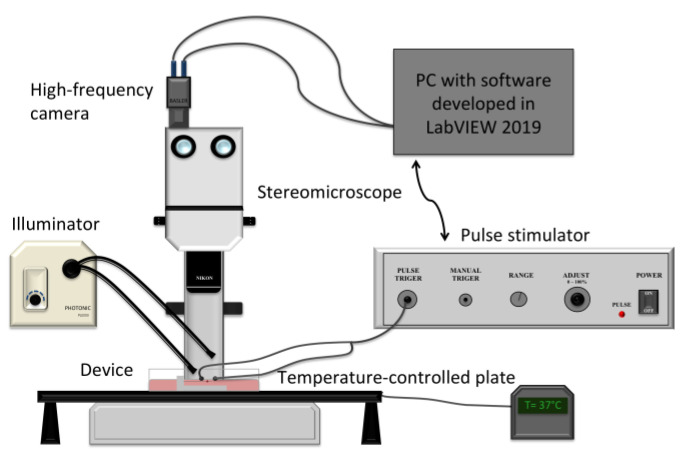
Schematic of the entire experimental setup used for the measurement of the contractile force of X-METs.

Figure 2 shows the proposed sensor integrated with the platinum electrodes for the tissue’s stimulation. The two custom-built platinum electrodes were placed parallel to the tissue and connected to a pulse stimulator (Aurora Scientific Inc. 701C, Ontario, Canada) to elicit the tissue contraction. The entire sensor was placed under a stereomicroscope (SMZ 800, Nikon, Tokyo, Japan) equipped with a high-frequency camera (aca2040–180 km, Basler, Ahrensburg, Germany) for image acquisition, and an illuminator (Photonic PL-3000, Coquitlam, Canada) was employed to adjust the image contrast for the post-processing correlation of the images, as shown in the schematic of the entire experimental setup in Figure 3.

**Figure 4:**
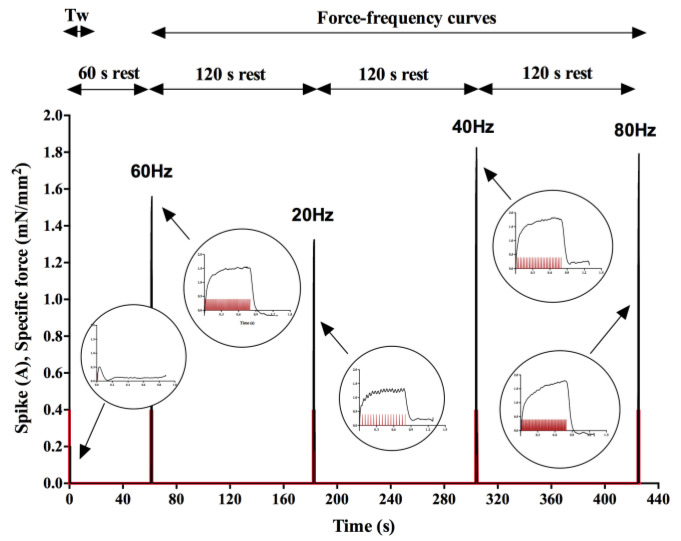
Example of an entire stimulation protocol for the measurement of the contractility of a control X-MET on day 0 of acquisition. Specific force (black) developed by the tissue when subjected to one twitch test and four tetanic stimulations (current spikes are in red).

The stimulation protocol was constituted of one twitch test and four unfused/fused tetanic stimulations, in isometric conditions, to measure tissue contractile kinetics and the force–frequency relationship, respectively. Figure 4 shows an example of an entire stimulation protocol for a control X-MET on the first day of acquisition. All the stimulation parameters were chosen accordingly to those used in our previous works [13,14,15] and in the literature [21]. The current intensity of a single pulse was set to 400 mA for both twitch test and tetanic stimulations. The twitch response was obtained with a 1 ms single pulse stimulation. These parameters were shown to elicit the maximum twitch force (F_TW_) of the X-MET [13] in a standard supramaximal way (i.e., +50% of the maximum current value eliciting maximum twitch force). The four tetanic stimulations were delivered in an alternate order, namely, at 60 Hz, 20 Hz, 40 Hz and 80 Hz, to avoid tissue adaptation to increasing or decreasing frequencies, with 1 ms pulse trains of a duration of 0.8 s. A resting time of 60 s was allowed before the first train stimulation, and 120 s of rest was allowed before the following ones. These values were chosen according to a series of preliminary experiments aimed at evaluating the minimum resting time necessary to avoid tissue fatigue [42,43,44].

**Figure 5:**
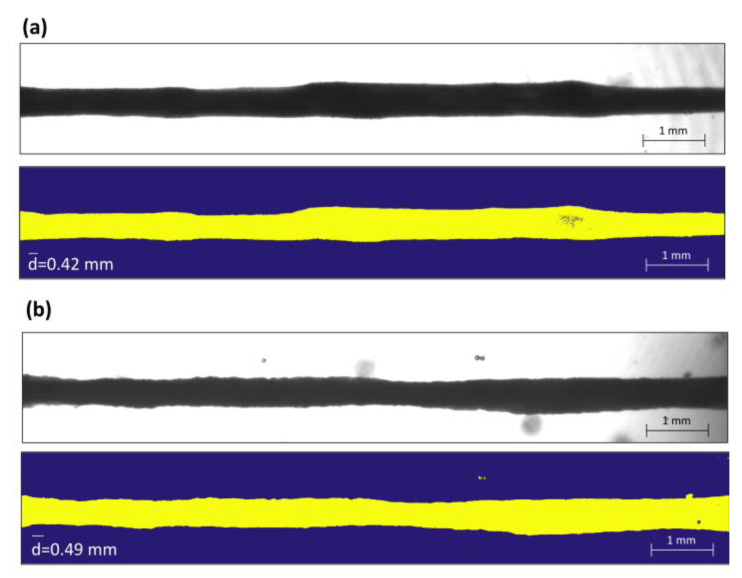
Example of two X-METs acquired for the calculation of the CSA. All the images were acquired at 1X magnification and a resolution of 2040 × 300 pixels. Top, image of the X-MET acquired with the camera; bottom, the same image after being processed with the Matlab program for the calculation of the average diameter, respectively for a control (**a**) and a treated (**b**) sample.

Time to peak (TTP) and half relaxation time (1/2RT) were measured from the twitch response to characterize the contractile kinetics of the tested X-METs. The active force generated during the twitch test and the tetanic stimulation was measured and divided by the tissue average cross-sectional area (CSA) to compute the specific force. The force–frequency curves were then obtained for the control and treated X-METs. For this, before each test, an image of the entire tissue was acquired to calculate the cross-sectional area for all the acquisition days. An example of two images acquired for the measurement of the CSA is shown in Figure 5, for a control and a treated sample. Each image was processed with a program developed ad hoc in Matlab. At first, a mask of the X-MET was obtained based on the evaluation of the grey levels of the images to identify the shape of the X-MET. After that, the program measured the average of all the diameters computed for each pixel along the tissue’s longitudinal axis, and a circular section was assumed to compute the CSA [27]. For the entire duration of the test, the tissue was maintained at a temperature of 37 ± 0.3 °C using a temperature control plate (H401, Okolabs.r.l., Naples, Italy).

Of note, since the aim of this work was to develop a sensor able to capture the defects in contractile capability of damaged tissues without affecting the correct development of healthy X-METs, we tested both untreated and treated tissues in parallel, i.e., originating from the same culture. This procedure was repeated for five different cell cultures with the aim of obtaining a better representation of the X-MET model. Since during one of the five cultures, a treated sample degraded before being clamped in the sensor, we reached a total number of five specimens for the control group and four specimens for the treated ones, and the values of the CSA, calculated on the first day of acquisition, were 0.10 ± 0.03 mm^2^ and 0.18 ± 0.12 mm^2^, respectively.

A software developed in LabVIEW 2019 allowed us to set all the test parameters as well as to synchronize the electric stimulation with the image acquisition. The images were acquired at 300 fps with an optical magnification of 2X and a resolution of 1020 × 300 pixels. This combination of parameters resulted in being the optimal working condition for the tracking algorithm in terms of accuracy in capturing the actual movement of the elastic pin [38].

### 2.4. Contraction Force Measurement through the Optical Tracking Method

**Figure 6:**
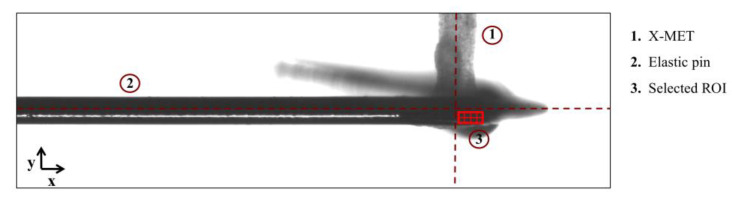
Example of an image acquired for the X-MET contractility measurement: magnification of 2X and resolution of 1020 × 300 pixels. The resulting ROI selected for image processing moved from the central axis between the X-MET and the elastic pin.

The images of the X-MET connected to the elastic pin were processed by an optical tracking method to measure the displacement of the pin tip when subjected to tissue contraction. The algorithm was developed in LabVIEW 2019 using IMAQ Optical Flow VI and based on the computation of the change in location of a set of featured points between two consecutive images [38,45]. For all the experimental tests, a region of interest (ROI) of 40 × 15 pixels was selected on the pin in correspondence with the engineered muscle tissue with the longer side parallel to the pin axis and divided into 15 nodes [41]. The tracking algorithm computed the average axial displacements (along *y* direction) between all the nodes included in the ROI. Figure 6 shows an example of the ROI positioning on the elastic pin in correspondence with the X-MET. It is important to remark that the ROI positioning was highly related to the contrast and illumination of the acquired images, and therefore, it was not always possible to place it in the exact axis center. To address this, in a previous work [41], we evaluated the error introduced in the correlation algorithm by a wrong positioning of the ROI, and all the displacement values here obtained from the experimental tests were therefore corrected to compensate for this error. The displacements obtained from the tracking algorithm were then converted in force values by multiplying them for the experimental elastic constant, and finally, they were divided by the *CSA* to obtain the specific forces:

(2) $$F_{specific}=\frac{\delta_{meas}*k}{CSA}$$

where *δ_meas_* is the displacement along the y direction measured by the tracking algorithm and *k* is the experimental elastic constant obtained through the elastic pin calibration.

### 2.5. Statistical Analysis

All the statistical analyses were carried out with GraphPad Prism 6.0, and differences were considered significant when *p* < 0.05.

The linear relationship between the force measured by the micro-force transducer and the displacement imposed by the linear actuator was assessed using linear regression with *p*-value fixed at 0.05 for the average measured force across the 10 repetitions for each displacement in input.

A one-way ANOVA, followed by multiple comparison, was performed to look for differences in all the measured parameters across the five days, i.e., assuming the *day* as the fixed factor, for the untreated group and for the specific twitch force and the maximum tetanic force for the treated group.

A two-way ANOVA was performed assuming the *day* and the *treatment* as fixed factors, followed by a multiple comparison to look for differences in the specific force at all the tested frequencies, including the twitch test. Of note, three treated tissues “almost died” during the accelerated aging treatment, and their generated force was therefore null for the remaining testing days. For these samples, it was not possible to measure TTP and 1/2RT on day 3 and day 4.

Values are expressed as mean ± SD.

## 3. Results

### 3.1. Force-Displacement Calibration

**Figure 7:**
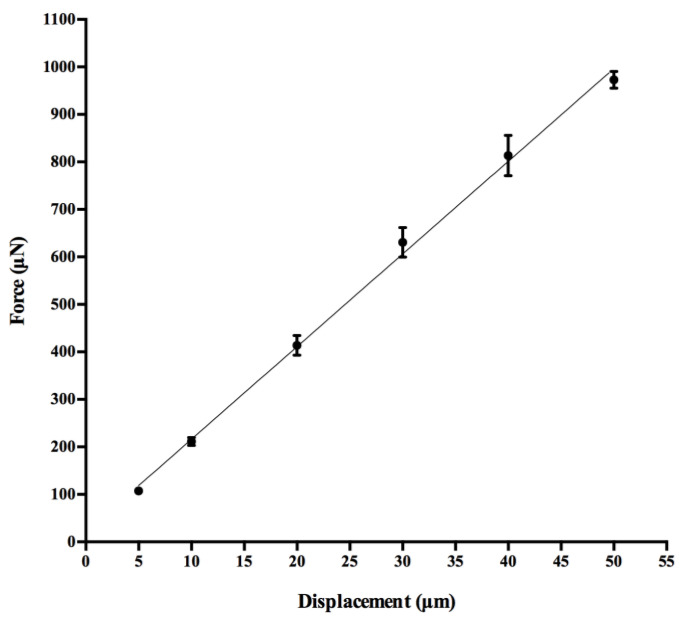
Linear regression for the force values measured by the micro-force transducer during the pin calibration procedure. For each displacement input, mean ± SD of the measured force are displayed.

The linear regression test performed for the elastic pin calibration is shown in Figure 7. The regression analysis showed R^2^ equal to 0.992, reflecting a highly linear relationship between the force developed by the elastic pin, at a length of L = 22.7 mm, measured by the micro-force transducer and the average displacement values imposed by the linear actuator. In addition, the coefficient of variation (CV) obtained for the force values was generally lower than 5% for all the imposed displacements. The experimental elastic constant, calculated as the slope of the curve, was k = 19.51 N/m, a value close to that obtained theoretically (20 N/m).

### 3.2. Contractility Measurements

**Figure 8:**
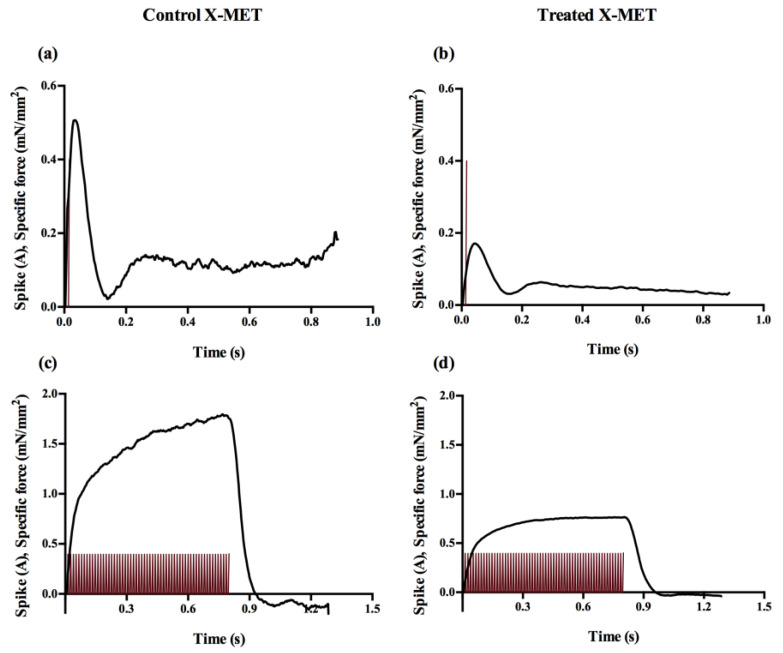
Example of the specific force developed by the X-MET on the first day of the experimental tests. Specific force (black) developed when subjecting the tissues to the twitch test (current spikes are in red) for control (**a**) and treated (**b**) X-MET and specific force developed during the 80 Hz pulse train (current spikes are in red) for control (**c**) and treated (**d**) X-MET.

Figure 8 shows an example of the specific force developed by a control and a treated X-MET when subjected to a twitch test and a pulse train of 80 Hz on the first day of acquisition. Interestingly, the force response measured through the optical tracking was highly consistent with that obtained with micro-force transducers [14,21,46], highlighting how this method was also able to follow dynamic changes in the input variable.

Table 1 summarizes all the parameters measured during the twitch test for both the control and treated X-METs across the five days of acquisition. The one-way ANOVA confirmed that no significant alterations occurred in TTP, 1/2RT or specific twitch force across the five testing days in the control group. On the other hand, the one-way ANOVA showed that the specific twitch force developed by the treated X-METs was significantly affected by the *day* factor, and the Tuckey’s multiple comparison tests revealed a significant decrease in force on day 2, day 3 and day 4 in comparison with day 0. The two-way ANOVA showed that both the *day* and *treatment* factors significantly affected the specific twitch force. Post hoc tests also highlighted significant differences between the control and treated X-METs on day 1, day 3 and day 4.

**Figure 9:**
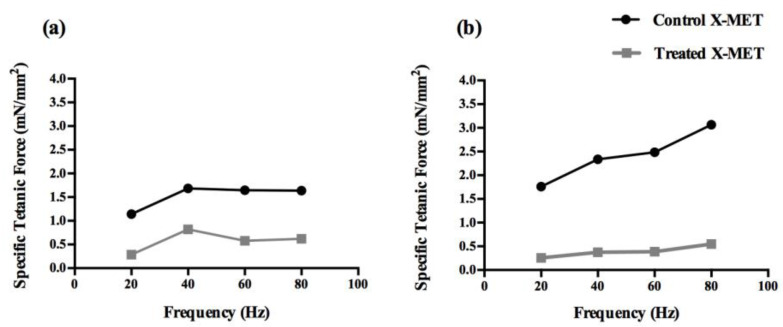
The force–frequency curves for one control and one treated X-MET obtained on day 0 (**a**) and day 4 (**b**) of acquisition.

Figure 9 shows an example of the force–frequency curves for a control and a treated X-MET on day 0 and day 4 of acquisition. The results show that on the first day of acquisition, the control and treated X-METs display similar specific tetanic forces. On the contrary, on day 4, the treated X-METs developed a specific tetanic force lower than that developed on day 0, and this outcome is representative of all the tested tissues. Moreover, on day 4 of acquisition, the maximum specific tetanic force developed by the engineered tissues occurred at a stimulation frequency of 80 Hz for both the control and treated X-METs.

**Figure 10:**
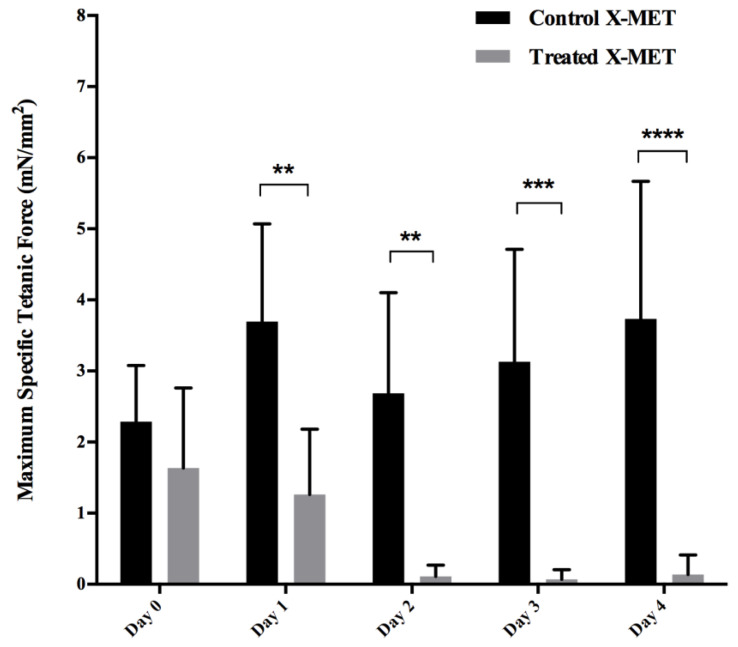
Maximum specific tetanic force in control and treated X-METs for the five days of acquisition. ** *p*-value < 0.01. *** *p*-value < 0.001. **** *p*-value < 0.0001 (from two-way ANOVA).

The maximum specific tetanic force developed by the control and treated X-METs across all the days is shown in Figure 10. The one-way ANOVA did not show any significant differences in the maximum specific force generated by control X-METs across the five days of experiment, confirming that the proposed sensor did not affect the tissue’s growth and maturation. On the other hand, the one-way ANOVA applied to the treated group revealed a significant influence of the *day* factor on the maximum specific force. The two-way ANOVA revealed that only the *treatment* factor significantly affected the maximum specific force. Fisher’s multiple-comparison test also showed significant decreases in the maximum specific tetanic forces developed by the treated X-METs when compared with those developed by the control specimens on all testing days except the initial one. This latter point also confirmed that the treated specimens were as good as the untreated ones before the beginning of the treatment.

## 4. Discussions

The aim of this work was to develop an innovative embedded sensor for the continuous, noninvasive, optical measurement of skeletal muscle engineered tissue’s contractility, whose loss could occur in several compromised conditions [47], through the deflection of a calibrated pin. The proposed sensor configuration allowed us to create a mechanical environment for the engineered muscle tissue very similar to that obtained during its normal growth, where the tissue was usually clamped between two fixed pins [13,15,48]. The coefficients of variation obtained during the calibration procedure confirmed the effectiveness of our sensor in the force measurements, and the results obtained testing five healthy X-METs showed that the sensor was able to follow the dynamic changes in the force responses without altering the growth and maturation of the engineered tissues.

In addition, the contractile force measured through the optical tracking method was highly consistent with that obtained for the X-METs with micro-force transducers in our previous studies [14,15]. At this point, it is important to remark that a correct comparison of the specific force values between different engineered muscle tissues can be performed only when different parameters are taken into account. First, it was shown that the technique used for the measurements of the contractility, like the cantilever method [46] or the force transducer [21], can influence the range of the measured force [49]. Moreover, other parameters like the time maturation, assessment time and the length and mass of the construct have to be taken in consideration for a correct comparison of the contractile forces. Finally, the methodology used to calculate the CSA has a crucial role in contractility measurements. In the literature, different techniques have been proposed for the measurement of the CSA [49] that differ in how they approximate the construct shape, such as circular, rectangular or elliptical. However, most of the works referred to a contractile force normalized to the whole CSA, calculated as a circular shape [49]. Of note, since engineered muscle tissues can significantly vary in dimension from one sample to another, it is crucial to report all the generated forces in terms of normalized values. Nonetheless, within this context, the results obtained in this work were of the same order of magnitude as that obtained by others in terms of maximum specific force [21,46,49].

Once we assessed that our sensor was able to measure the contractile force in an accurate and reproducible manner, we used it to discriminate a specific tissue culture condition in which the reduction of growth factors was able to mimic aged microenvironments. Our results showed that the treated X-METs displayed a decrease in specific force starting from the third day of acquisition. This outcome is in high accordance with the evidence reported in the literature, where significant skeletal muscle wasting associated with aging was shown to cause profound muscle weakness with a significant reduction in force production due to the decrease in muscle mass [39,40]. Regarding the specific twitch force, our results showed a significant decrease between the control and treated X-METs at day 1, day 3 and day 4, confirming the capacity of the proposed sensor to discriminate alterations in the force developed by those engineered tissues artificially treated to simulate an aged microenvironment, which in turn was shown to be a good model of accelerated aging also on 3D tissues.

Our results allowed us to obtain a force–frequency curve for the control X-METs highly similar to that of skeletal muscle [50], confirming that the proposed sensor did not affect the tissue’s growth and maturation and that the stimulation protocol we chose did not damage the tissues, preserving their contractility during the five days. On the contrary, the treated X-METs showed significantly lower force values on the last day of acquisition. Of note, both the control and treated X-METs showed a tetanic frequency equal to 80 Hz. This outcome was in agreement with a previous work [13] with healthy tissues. On the other hand, for treated tissues, this result could suggest that no significant changes occurred in the tissue composition during the measurement of their contractility. However, this hypothesis has to be confirmed with a series of future histo-morphological and metabolic analyses. Moreover, the maximum specific tetanic force obtained for the control X-METs showed almost constant values across all the days, in accordance with the literature [10]. On the contrary, significant differences in the maximum specific force were obtained for the treated X-METs as the days increased. This confirmed the ability of the proposed sensor to capture alterations in the contractile capability of engineered muscle tissues. Typically, the engineered muscle tissues demonstrated a possible adaptation to the clamping in the sensor, showing an increase in the contractile force on day 1 with respect to day 0 for both the control and treated X-METs.

Since recently, tissue engineering approaches have been proposed for the design of *in-vitro* models of healthy or pathological tissues and organs that can be employed for drug screening and the evaluation of new therapies, the use of an embedded platform like the one here devised could provide important evidence in the study of different physio-pathological conditions [51,52] characterized by alterations in contractile force [8,9,53]. In view of this, we propose a sensor’s applicability in the monitoring of engineered muscle tissue’s functional properties to identify a specific pharmacological treatment or a pathological condition.

## 5. Conclusions

In this study, we developed an innovative sensor for *in-vitro* measurements of engineered muscle tissue contractility based on the detection of the displacement of an elastic pin caused by the tissue contraction through an optical tracking method. The sensor was designed to not alter the engineered tissue’s growth and development, being fully embedded in standard culture dishes. To allow the measurements of the contractility in a noninvasive manner, the sensor was integrated with an optical setup that performed continuous measurement for a long period. The setup also integrated two platinum electrodes connected to a pulse stimulator to deliver specific electric stimulation protocols. First, the sensor was calibrated to associate the displacement values to the force ones with the use of a step linear motor and a micro-force transducer. Then, it was used for the measurement of the force of an engineered muscle tissue, named X-MET, under a specific electric stimulation protocol for five consecutive days. The experimental results confirmed that the proposed sensor was able to accurately measure the tissue contraction without affecting the tissue’s growth and maturation. In parallel, the sensor was used to measure the contractile force occurring in a particular physio-pathological condition, accelerated aging. The results clearly showed that the sensor was also able to discriminate alterations in the force developed by the tissues treated to simulate an aged microenvironment.

In conclusion, we here proposed an innovative sensor based on a noninvasive method for the measurement of engineered tissues’ contractility to be integrated in a platform to elucidate functional changes in all diseases in which muscle contractility could be altered, both as a spontaneous contraction and following electrical stimulation.

## Figures and Tables

**Table 1:** Kinetic contractile parameters (TTP and 1/2RT) and specific twitch force (F_TW_/CSA) in control and treated X-METs for the five days of acquisition. Values are mean ± SD. n = 5 for control X-MET, n = 4 for treated X-MET. *** *p*-value < 0.001, ** *p*-value < 0.01 vs. treated X-METs (from two-way ANOVA). ^+^ *p*-value < 0.05 vs. day 0 (from one-way ANOVA).

	TTP (ms)		1/2RT (ms)		F_TW_/CSA (mN/mm^2^)	
	Control	Treated	Control	Treated	Control	Treated
**Day 0**	40.7 ± 8.9	42.5 ± 5.0	59.3 ± 18.2	57.5 ± 12.6	0.7 ± 0.5	0.5 ± 0.3
**Day 1**	34.0 ± 8.0	32.2 ± 1.9	70.0 ± 9.4	45.0 ± 16.6	1.6 ± 0.7 ***	0.3 ± 0.3
**Day 2**	47.3 ± 13.2	35.0 ± 16.6	72.0 ± 16.8	70.0 ± 18.8	0.8 ± 0.6	0.1 ± 0.1 ^+^
**Day 3**	41.3 ± 10.7	50.0	64.7 ± 16.4	73.3	1.1 ± 0.5 **	0.0 ± 0.1 ^+^
**Day 4**	37.7 ± 18.2	13.3	64.0 ± 17.5	16.7	1.3 ± 0.5 **	0.1 ± 0.1 ^+^

## Data Availability

Not applicable.

## References

[1] Mao A.S., Mooney D.J. (2015). Regenerative medicine: Current therapies and future directions. Proc. Natl. Acad. Sci. USA.

[2] Dzobo K., Thomford N.E., Senthebane D.A., Shipanga H., Rowe A., Dandara C., Pillay M., Motaung K.S.C.M. (2018). Advances in regenerative medicine and tissue engineering: Innovation and transformation of medicine. Stem Cells Int..

[3] Olson J.L., Atala A., Yoo J.J. (2011). Tissue Engineering: Current Strategies and Future Directions. Chonnam Med. J..

[4] Boschetti F. (2022). Tissue Mechanics and Tissue Engineering. Appl. Sci..

[5] Karagounis L.G., Hawley J.A. (2010). Skeletal muscle: Increasing the size of the locomotor cell. Int. J. Biochem. Cell Biol..

[6] Liu G., Mac Gabhann F., Popel A.S. (2012). Effects of Fiber Type and Size on the Heterogeneity of Oxygen Distribution in Exercising Skeletal Muscle. PLoS ONE.

[7] Kang M.S., Lee S.H., Park W.J., Lee J.E., Kim B., Han D.W. (2020). Advanced techniques for skeletal muscle tissue engineering and regeneration. Bioengineering.

[8] Guettier-Sigrist S., Coupin G., Braun S., Warter J.M., Poindron P. (1998). Muscle could be the therapeutic target in SMA treatment. J. Neurosci. Res..

[9] Khodabukus A., Kaza A., Wang J., Prabhu N., Goldstein R., Vaidya V.S., Bursac N. (2020). Tissue-engineered human myobundle system as a platform for evaluation of skeletal muscle injury biomarkers. Toxicol. Sci..

[10] Khodabukus A. (2021). Tissue-Engineered Skeletal Muscle Models to Study Muscle Function, Plasticity, and Disease. Front. Physiol..

[11] Kim J., Kong J.S., Han W., Kim B.S., Cho D.W. (2020). 3d cell printing of tissue/organ-mimicking constructs for therapeutic and drug testing applications. Int. J. Mol. Sci..

[12] Bédard P., Gauvin S., Ferland K., Caneparo C., Pellerin È., Chabaud S., Bolduc S. (2020). Innovative human three-dimensional tissue-engineered models as an alternative to animal testing. Bioengineering.

[13] Carosio S., Barberi L., Rizzuto E., Nicoletti C., Del Prete Z., Musarò A. (2013). Generation of eX vivo-vascularized Muscle Engineered Tissue (X-MET). Sci. Rep..

[14] Rizzuto E., Carosio S., Faraldi M., Pisu S., Musarò A., Del Prete Z. (2016). A DIC Based Technique to Measure the Contraction of a Skeletal Muscle Engineered Tissue. Appl. Bionics Biomech..

[15] Pisu S., Cosentino M., Apa L., Musarò A., Rizzuto E., Del Prete Z. (2019). Measuring the Maximum Power of an ex vivo Engineered Muscle Tissue with Isovelocity Shortening Technique. IEEE Trans. Instrum. Meas..

[16] Pisu S., Apa L., Cosentino M., Musarò A., Rizzuto E., Del Prete Z. Measuring the X-MET’s maximum power: A preliminary study. Proceedings of the MeMeA 2018 IEEE International Symposium on Medical Measurements and Applications.

[17] Alave Reyes-Furrer A., De Andrade S., Bachmann D., Jeker H., Steinmann M., Accart N., Dunbar A., Rausch M., Bono E., Rimann M. (2021). Matrigel 3D bioprinting of contractile human skeletal muscle models recapitulating exercise and pharmacological responses. Commun. Biol..

[18] Turner M.C., Rimington R.P., Martin N.R.W., Fleming J.W., Capel A.J., Hodson L., Lewis M.P. (2021). Physiological and pathophysiological concentrations of fatty acids induce lipid droplet accumulation and impair functional performance of tissue engineered skeletal muscle. J. Cell. Physiol..

[19] Sasaki D., Matsuura K., Seta H., Haraguchi Y., Okano T., Shimizu T. (2018). Contractile force measurement of human induced pluripotent stem cell-derived cardiac cell sheet-tissue. PLoS ONE.

[20] Juhas M., Bursac N. (2014). Roles of adherent myogenic cells and dynamic culture in engineered muscle function and maintenance of satellite cells. Biomaterials.

[21] Rao L., Qian Y., Khodabukus A., Ribar T., Bursac N. (2018). Engineering human pluripotent stem cells into a functional skeletal muscle tissue. Nat. Commun..

[22] Dennis R.G., Kosnik P.E., Gilbert M.E., Faulkner J.A. (2001). Excitability and contractility of skeletal muscle engineered from primary cultures and cell lines. Am. J. Physiol.-Cell Physiol..

[23] Xu B., Zhang M., Perlingeiro R.C.R., Shen W. (2019). Skeletal Muscle Constructs Engineered from Human Embryonic Stem Cell Derived Myogenic Progenitors Exhibit Enhanced Contractile Forces When Differentiated in a Medium Containing EGM-2 Supplements. Adv. Biosyst..

[24] Wilson K., Das M., Wahl K.J., Colton R.J., Hickman J. (2010). Measurement of contractile stress generated by cultured rat muscle on silicon cantilevers for toxin detection and muscle performance enhancement. PLoS ONE.

[25] Santoso J.W., Li X., Gupta D., Suh G.C., Hendricks E., Lin S., Perry S., Ichida J.K., Dickman D., McCain M.L. (2021). Engineering skeletal muscle tissues with advanced maturity improves synapse formation with human induced pluripotent stem cell-derived motor neurons. APL Bioeng..

[26] Guo X., Badu-Mensah A., Thomas M.C., McAleer C.W., Hickman J.J. (2020). Characterization of functional human skeletal myotubes and neuromuscular junction derived-from the same induced pluripotent stem cell source. Bioengineering.

[27] Nagashima T., Hadiwidjaja S., Ohsumi S., Murata A., Hisada T., Kato R., Okada Y., Honda H., Shimizu K. (2020). In Vitro Model of Human Skeletal Muscle Tissues with Contractility Fabricated by Immortalized Human Myogenic Cells. Adv. Biosyst..

[28] Rausch M., Böhringer D., Steinmann M., Schubert D.W., Schrüfer S., Mark C., Fabry B. (2020). Measurement of Skeletal Muscle Fiber Contractility with High-Speed Traction Microscopy. Biophys. J..

[29] Pasqualini F.S., Agarwal A., O’Connor B.B., Liu Q., Sheehy S.P., Parker K.K. (2018). Traction force microscopy of engineered cardiac tissues. PLoS ONE.

[30] Pasquarelli A. (2021). Microelectrode Arrays, Implants, and Organs-on-a-Chip. Biosens. Biochips.

[31] Sala L., Van Meer B.J., Tertoolen L.G.J., Bakkers J., Bellin M., Davis R.P., Denning C., Dieben M.A.E., Eschenhagen T., Giacomelli E. (2018). Musclemotion: A versatile open software tool to quantify cardiomyocyte and cardiac muscle contraction in vitro and in vivo. Circ. Res..

[32] Bruegmann T., Malan D., Hesse M., Beiert T., Fuegemann C.J., Fleischmann B.K., Sasse P. (2010). Optogenetic control of heart muscle in vitro and in vivo. Nat. Methods.

[33] Francis G.L. (2010). Albumin and mammalian cell culture: Implications for biotechnology applications. Cytotechnology.

[34] Ham R.G. (1963). Albumin replacement by fatty acids in clonal growth of mammalian cells. Science.

[35] Baumgartner R.N., Koehler K.M., Romero L., Garry P.J. (1996). Serum albumin is associated with skeletal muscle in elderly men and women. Am. J. Clin. Nutr..

[36] Moustogiannis A., Philippou A., Taso O., Zevolis E., Pappa M., Chatzigeorgiou A., Koutsilieris M. (2021). The effects of muscle cell aging on myogenesis. Int. J. Mol. Sci..

[37] Dennis R.G., Kosnik P.E. (2000). Excitability and isometric contractile properties of mammalian skeletal muscle constructs engineered in vitro. Vitr. Cell. Dev. Biol.-Anim..

[38] Apa L., Martelli F., Rizzuto E., Del Prete Z. Design of a new device to measure skeletal muscle engineered tissues’ contractile force by using an optical tracking technique. Proceedings of the Medical Measurements and Applications, MeMeA.

[39] Larsson L., Degens H., Li M., Salviati L., Lee Y.I., Thompson W., Kirkland J.L., Sandri M. (2019). Sarcopenia: Aging-related loss of muscle mass and function. Physiol. Rev..

[40] McCormick R., Vasilaki A. (2018). Age-related changes in skeletal muscle: Changes to life-style as a therapy. Biogerontology.

[41] Forconi F., Apa L., Marianna C., Antonio M., Emanuele R., Zaccaria D.P. Effects of ROI positioning on the measurement of engineered muscle tissue contractility with an optical tracking method. Proceedings of the 2022 IEEE International Symposium on Medical Measurements and Applications (MeMeA).

[42] Forconi F., Apa L., Pisu S., Casola I., Musarò A., Rizzuto E., Del Prete Z. (2022). Development of a Novel Technique for the Measurement of Neuromuscular Junction Functionality in Isotonic Conditions. Cell. Mol. Bioeng..

[43] Del Prete Z., Musarò A., Rizzuto E. (2008). Measuring mechanical properties, including isotonic fatigue, of fast and slow MLC/mIgf-1 transgenic skeletal muscle. Ann. Biomed. Eng..

[44] Pisu S., Rizzuto E., Musaro A., Del Prete Z. Identification of the best stimulation parameters to measure in situ the comunication between muscle and nerve in mouse Tibialis muscle. Proceedings of the 2017 IEEE International Symposium on Medical Measurements and Applications, MeMeA.

[45] Horn B.K.P., Schunck B.G. (1981). Determining optical flow. Artif. Intell..

[46] Cheng C.S., Davis B.N.J., Madden L., Bursac N., Truskey G.A. (2014). Physiology and metabolism of tissue-engineered skeletal muscle. Exp. Biol. Med..

[47] Qazi T.H., Mooney D.J., Pumberger M., Geißler S., Duda G.N. (2015). Biomaterials based strategies for skeletal muscle tissue engineering: Existing technologies and future trends. Biomaterials.

[48] Rizzuto E., Carosio S., Musaro A., Del Prete Z. A Digital Image Correlation based technique to control the development of a skeletal muscle engineered tissue by measuring its surface strain field. Proceedings of the 2015 IEEE International Symposium on Medical Measurements and Applications, MeMeA.

[49] Vesga-Castro C., Aldazabal J., Vallejo-Illarramendi A., Paredes J. (2022). Contractile force assessment methods for in vitro skeletal muscle tissues. Elife.

[50] Lee S.C.K., Russ D.W., Binder-Macleod S.A. (2008). Force-Frequency Relation of Skeletal Muscle. Encyclopedia of Neuroscience.

[51] Shimizu K., Genma R., Gotou Y., Nagasaka S., Honda H. (2017). Three-dimensional culture model of skeletal muscle tissue with atrophy induced by dexamethasone. Bioengineering.

[52] Vandenburgh H., Shansky J., Benesch-Lee F., Barbata V., Reid J., Thorrez L., Valentini R., Crawford G. (2008). Drug-screening platform based on the contractility of tissue-engineered muscle. Muscle Nerve.

[53] Urciuoli E., Peruzzi B. (2020). Involvement of the fak network in pathologies related to altered mechanotransduction. Int. J. Mol. Sci..

